# The role of the social vulnerability index in personal protective equipment shortages, number of cases, and associated mortality during the coronavirus disease 2019 (COVID-19) pandemic in Michigan skilled nursing facilities

**DOI:** 10.1017/ice.2020.1318

**Published:** 2020-11-13

**Authors:** Jennifer J. LeRose, Courtney Merlo, Phong Duong, Kelsi Harden, Rebecca Rush, Adam Artzberger, Nikki Sidhu, Avnish Sandhu, Teena Chopra

**Affiliations:** 1 Michigan State University College of Osteopathic Medicine, East Lansing, Michigan; 2 Wayne State University School of Medicine, Detroit, Michigan; 3 Division of Infectious Diseases, Department of Internal Medicine, Detroit Medical Center, Wayne State University School of Medicine, Detroit, Michigan

## Abstract

The Social Vulnerability Index (SVI) is used to stratify community need for support during disasters. We evaluated relationships between the SVI and personal protective equipment shortages, COVID-19 caseload, and mortality rates in skilled nursing facilities (SNFs). In SVI quartile 4, personal protective equipment shortages were 2.3 times those in SNFs in quartile 1; COVID-19 case loads were 1.6 times those of SNFs in quartile 1; and mortality rates in were 1.9 times those of SNFs in SVI quartile 1.

Emergency preparedness plans must be established by communities to prepare for and respond to hazardous events. Such plans depend upon numerous components including socioeconomic status, disability, and transportation. These social determinants of health are among 15 variables used by the Centers for Disease Control and Prevention to calculate the Social Vulnerability Index (SVI), a tool used to identify communities that require additional support during disasters, such as the coronavirus disease 2019 (COVID-19) pandemic.^1^

COVID-19 transmission occurs primarily through respiratory droplets, underscoring the importance of protecting healthcare workers (HCWs) from disease through proper use of personal protective equipment (PPE).^2^ From May to July 2020, 1 in 5 skilled nursing facilities (SNFs) in the United States reported PPE shortages.^4^ Due to resource deficits and a broken public health infrastructure, SNFs were forced to implement unconventional infection control strategies inconsistent with typical US standards, increasing HCW exposure risk.^3^ To date, Michigan SNFs have reported 8,775 confirmed COVID-19 cases and 2,180 associated deaths, accounting for 7% of total cases and 32% of deaths.^4^

Data that explore the relationship between the SVI and pandemic-related infection control metrics are limited. Our primary objective was to determine whether a correlation existed between the SVI and PPE supply shortages in Michigan SNFs. Additionally, we analyzed the potential relationship between the SVI and the number of COVID-19 cases and mortality rate.

## Methods

### Study design

We conducted a retrospective, cross-sectional study focusing on SNFs in the Detroit metropolitan area to examine the relationship between the SVI and PPE supply, number of COVID-19 cases, and mortality rates. The SVI ranks communities by quartiles based on social determinants of health and available resources. Quartile 1 is considered least vulnerable while quartile 4 is most vulnerable.^1^ Data were collected in May 2020. We obtained approval for the study from our institutional review board.

### Data collection

Leadership at SNFs with ≥20 residential COVID-19 cases and/or facilities that reported COVID-19 infections in HCWs were contacted by phone to complete a standardized needs assessment. The survey collected data regarding number of staff with direct patient contact, PPE supply, use and reuse policies, and estimated arrival of next PPE shipment. PPE shortage was defined as a facility’s inability to meet the usual standard of care in protecting HCWs between the time of the interview and the arrival of the next PPE shipment. The number of COVID-19 cases and deaths by facility were collected from the Michigan Department of Health and Human Services.^4^

### Statistical analysis

The SNFs were divided into cohorts based on SVI quartile.^5^ The numbers of COVID-19 cases and deaths were normalized by the number of licensed beds. The Fisher exact χ^2^ test and Wilcoxon signed rank-sum test were used in the analysis. Categorical variables were reported as frequencies and percentages, and continuous variables were reported as means with standard deviations. Crude linear regression was completed for PPE shortages, number of cases, and mortality. *P* < .05 was considered statistically significant. SAS software (SAS Institute, Cary, NC) was used for the analysis.

## Results

### Personal protective equipment requests

In total, 124 SNFs were contacted for needs assessments, and 103 (83.1%) agreed to an interview. Also, 79.6% of these SNFs were located in the metropolitan area of Detroit. Overall, PPE items with the greatest shortages were gowns (82.5%), respirators (N95 or equivalent) (58.2%), and surgical masks (31.1%) (Table 1). Facilities ranked in SVI quartile 1 needed an average of 1.37 ± 1.36 PPE items. However, SNFs ranked in quartile 4 needed an average of 3.15 ± 1.37 PPE items (*P* = .006) (Table 1). For every SVI quartile increase, SNFs experienced 0.55 additional item shortages (R^2^ = 0.96).

**Table 1.** Personal Protective Equipment (PPE) Items Needed by Skilled Nursing Facilities, COVID-19 Case Burden, and Mortality Stratified by Social Vulnerability Index (SVI) Quartile

Variable	SVI 1 (n=11)	SVI 2 (n=25)	SVI 3 (n=33)	SVI 4 (n=34)	*P* Value^a^
**PPE item in shortage, no. (%)**					
Respirators	4 (36.4)	9 (36.0)	20 (60.6)	27 (79.4)	**.003**
Surgical mask	1 (9.1)	4 (16.0)	13 (39.4)	14 (41.2)	.055
Gowns	7 (63.6)	22 (88.0)	23 (69.7)	33 (97.1)	**.002**
Hand sanitizer	0 (0.0)	4 (16.0)	5 (15.2)	3 (8.8)	.587
Disinfectant	0 (0.0)	1 (4.0)	3 (9.1)	1 (2.9)	.676
Gloves	1 (9.1)	5 (20.0)	4 (12.1)	8 (23.5)	.562
Shoe covers	0 (0.0)	3(12.0)	6 (18.2)	3 (8.8)	.484
Shields	1 (9.1)	4 (16.0)	6 (18.2)	16 (47.1)	**.011**
Surgery caps	1 (9.1)	4 (16.0)	2 (6.1)	2 (5.9)	.531
No. of items requested, mean±SD	1.37±1.36	2.24±1.30	2.44±1.60	3.15±1.37	**.006**
No. of cases per beds, mean±SD	0.26±0.24	0.34±0.30	0.39±0.31	0.42±0.16	**.018**
No. of deaths per beds, mean±SD	0.07±0.04	0.08±0.06	0.09±0.07	0.13±0.08	**.016**

Note. SD, standard deviation.

a Bold *P* values indicate statistical significance at *P* < .05.

Items demonstrating statistically significant differences between SVI strata were respirators (*P* = .003), gowns (*P = .*002), and face shields (*P = .*011) (Table 1). Despite no statistical significance, surgical mask supply shortages also demonstrated incremental increases in the SVI quartile (*P* = .055) (Table 1). No correlation was detected between the SVI and the supplies of hand sanitizer, disinfectant, shoe covers, or surgical caps (Table 1).

### Number of COVID-19 cases and mortality

**Fig. 1.**
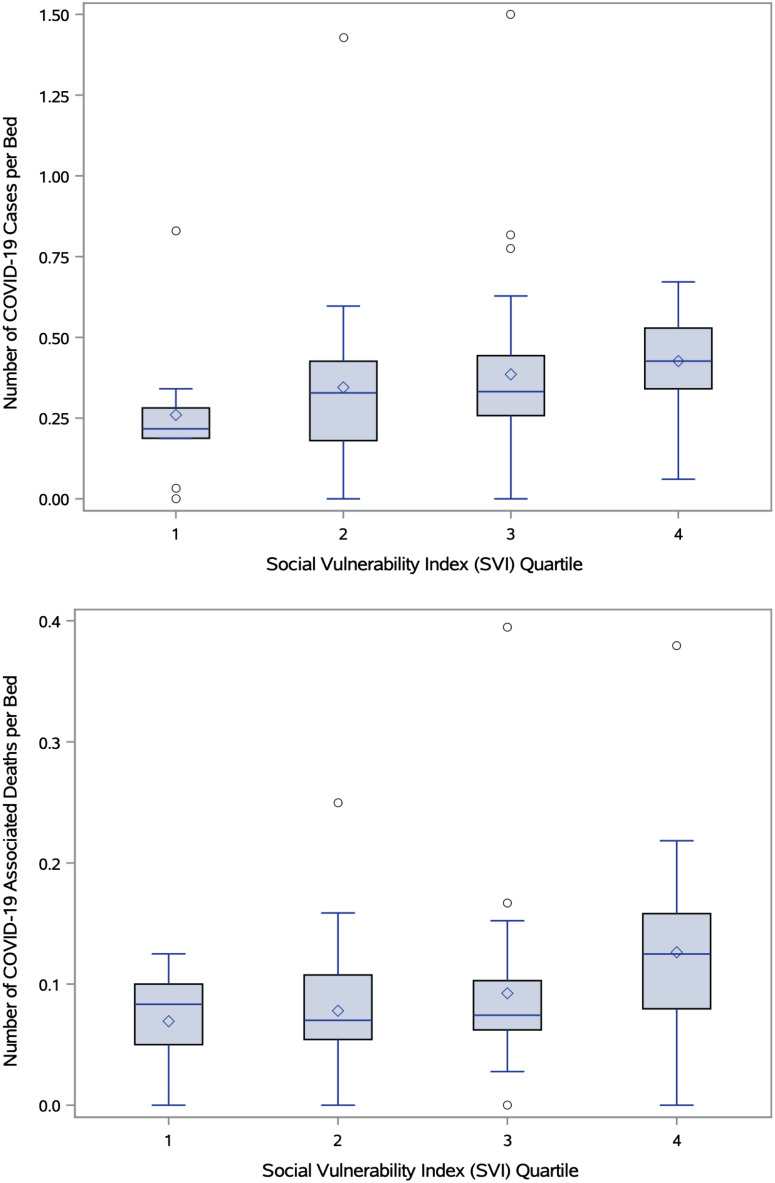
Box-plot diagram showing (top) number of COVID-19 cases and (bottom) COVID-19 mortality rates at skilled nursing facilities normalized to licensed bed number by Social Vulnerability Index (SVI) quartile. The boxes signify interquartile range, the median is represented by a short black line within the box, diamonds represent mean, and outliers are denoted by circles. Both number of COVID-19 cases and associated mortality have a significant correlations with the SVI at *P* < .05.

The number of COVID-19 cases and deaths per bed directly correlated with SVI quartiles. On average, SNFs had 0.37 ± 0.26 cases per bed. SNFs ranked in SVI quartile 1 had an average of 0.26 ± 0.24 cases per bed, whereas SNFs ranked in SVI quartile 4 averaged 0.42 ± 0.16 cases per bed, 1.62 times the number of cases seen in quartile 1 (*P* = .018) (Fig. 1).

Overall, the average mortality rate was 0.09 ± 0.07 per bed. The average mortality rate of SNFs in SVI quartile 4 was 0.13 ± 0.08 per bed (*P* = .016), compared to SNFs in SVI quartile 1, which averaged 0.07 ± 0.04 deaths per bed (Fig. 1). This mortality rate is 1.86 times greater for SNFs in SVI quartile 4 relative to those in quartile 1 (Table 1). With each SVI quartile increase, there was an increase of 0.05 cases and 0.02 associated deaths per bed (R^2^ = 0.96 and 0.87, respectively).

## Discussion

The data suggest that SNFs ranked in higher SVI quartiles experienced greater PPE shortages during the COVID-19 pandemic; each increase in SVI quartile correlated with 0.55 additional PPE item shortages. This disparity is likely due to reduced financial resources and greater reliance on government payers because SNFs in the highest quartile contain a greater proportion of Centers for Medicare & Medicaid Services (CMS) beds.^6^ Additionally, SNFs with a large population of Medicaid beds and more COVID-19 cases per licensed bed were more likely to report shortages.^2^ As a result, these facilities were likely outcompeted within the PPE marketplace, thus generating greater shortages.^7^

The number of COVID-19 cases and deaths per bed also increased with SVI quartile. An increase of 1 SVI quartile correlated to 0.05 more COVID-19 cases and 0.2 additional deaths per bed (R^2^ = 0.96 and 0.87, respectively). Although patient demographics and staffing shortages likely had the most significant impact on these outcomes, supply shortages presumably played an important role as well. PPE shortages likely led to breaches in infection control practices which, in turn, increased risk of viral transmission and subsequent infection.^8^ Therefore, inadequately protected HCWs may have acted as vectors to spread disease to other residents.^9^

Our study has several limitations. Information was not gathered regarding resident age, comorbidities, or other factors that contribute to increased COVID-19 susceptibility and mortality.^10^ Additionally, facilities did not provide information regarding their PPE supply chain operations. Therefore, we were unable to decipher if lack of supply was directly related to purchasing difficulties or delayed transit times on orders.^7^ Lastly, 21 facilities declined to complete a needs assessment. Of these, nearly half (n = 10) were in quartile 1. Consequentially, our sample size for the least vulnerable communities was relatively small.

The SNFs located in more vulnerable areas demonstrated an increased need for support during emergencies, indicated by greater PPE shortages and worse clinical outcomes during the COVID-19 pandemic. Therefore, agencies should prioritize supplying aid to the most vulnerable communities during crises to help minimize the disease spread and mortality rate exacerbated by health disparities.

## Supplementary material

For supplementary material accompanying this paper visit https://doi.org/10.1017/ice.2020.1318. click here to view supplementary material
